# Small scale lowpass and quad-band bandpass filter for 5G application

**DOI:** 10.1038/s41598-025-25757-z

**Published:** 2025-11-25

**Authors:** Dilip Kumar Choudhary, Kushagra Pratap

**Affiliations:** School of Electronics Engineering, Vellore Institute of Technology, Vellore, 632014 India

**Keywords:** Lowpass filter, Quad-band bandpass filter, Rectangular ring, Meander line, Energy science and technology, Engineering

## Abstract

This work offers a new small-scaled lowpass and quad-band passband filter with low insertion loss and high selectivity. By integrating a shorted interdigital capacitor (IDC), closed ring resonator, meander line, and rectangular-shaped virtual ground plane, better impedance matching and transmission properties have been attained. Initially, the lowpass response structure comprises shorted IDC and closed ring resonators. Further utilizing pairs of meander lines and rectangular stubs on both sides of ring resonators, a quad bandpass along with a lowpass filter was constructed. Excluding the fifty-ohm feedline the proposed filter area measures only 0.04*λ*_*g*_ × 0.06*λ*_*g*_ (15.6 mm×24.6 mm) at the lowpass frequency of 3dB cutoff, 0.75 GHz. The fractional bandwidth at 3-dB of the proposed four passband zones are 63.76% (1.39–2.78 GHz), 22.55% (3.26–4.09 GHz), 17.15% (4.42–5.24 GHz), and 4.3% (5.64–5.89 GHz) at operating frequencies of 02.18, 3.68, 4.78 and 5.82 GHz, respectively. Moreover, the resonance frequencies of each band can be adjusted by altering the structural parameters of the intended filter, which results in modifications to their relative lumped parameters. By doing experimental measurements that correlate with simulated ones, the suggested filter is validated. The designed filter may be useful for 5G communication frequency range-1 (FR1), 0.45–6.0 GHz bands.

## Introduction

With the speedy advancement of advanced wireless technologies over the last decade, there has been a significant growth in the need for new filters that can provide many services like lowpass, bandpass, and bandstop together. To achieve a small-scale microwave filter with low loss of insertion loss, multiple passband capability, good attenuation at band rejection, improved roll-off features, and comfort of incorporation, research is being concentrated on this goal^1^. A lowpass and bandpass (LP-BP) filter can separate signals using lowpass passbands such as direct current, intermediate frequency, and low-frequency baseband, and bandpass passbands such as local oscillator frequency and other useful radio-frequency signals. This filter can be used in mixers^2^. It is significant to remember that in mixer systems, splitting current, the local frequency of the oscillator, and transitional frequency require the use of a lowpass-bandpass combination^3^. Lowpass-bandpass filters are also necessary for radio arrangements with a mixture of optical fiber-coaxial links because baseband and microwave signals are sent over the same channel^4^. The lowpass filter with extensive band reject response was designed with the help of dual composite right and left-handed (DCRLH) resonators^5^. The structure comprises a shorted interdigital capacitor and rectangular stub to provide compactness and wider rejection with a high roll of factor. Shorted meandered lines and metamaterial structures are used to present a wideband band-pass filter^6^. Various multi-band bandpass filters like dual/triple/quad/penta passband were presented in^7^, having better electrical characteristics. Various techniques have been utilized to report this work. Grounded on waveguide technology, a small, double-layer, arrangeable tri-passband filter is investigated in^8^, with a broad bandwidth, compact design, and improved insertion loss.

Numerous filters by lowpass, multi-band passband filtering features have been reported. However, there are very few articles described in the literature that can produce different kinds of filtering responses at the same time, such as lowpass with single passband and dual and triple passband characteristics. Two filters that combine a dual-mode microstrip passband configuration with a stepped impedance lowpass filter architecture are proposed in^9^ to offer lowpass and passband filtering characteristics. The design of a diplexer with a lowpass-passband uses a straightforward matching circuit, although the bandpass band insertion loss is rather significant at 2.42 dB. Stepped impedance stubs were utilized in the design of lowpass and dual bandpass triplexers^10^. Selectivity and impedance matching were enhanced by the use of defective ground structures. In^11^, a two-working band as a lowpass and passband filter using quasi-lumped element resonators was introduced. By altering its physical dimensions, this configuration produces a small filter architecture that can separately adjust the frequencies. The multiple slots having hairpin shapes carved in the bottom of the structure are used in the literature^12^ to create lowpass and bandpass microstrip filters. It has a broad stopband and a readily adjustable center frequency that may be achieved by adjusting the hairpin slot’s length and breadth. Low pass filtering response along with dual pass band characteristics reported in^13^. It utilizes a coupled line folded structure and open stub to provide adjustable transmission zeros and operating frequencies. A different structure is used to suggest a miniaturized tri-band lowpass–bandpass filter. The first passband and the lowpass passband (LP) are created using transmission zeros and the second passband by lumped circuit^2^. Then the shunt stubs were added to the design to enhance stopband attenuation^14^. discusses another small, low-insertion-loss diplexer having low and band pass response for Wi-MAX applications. A miniaturized lowpass-dual pass band diplexer has been designed using meander line and ring resonators to provide high isolation between ports^15^. The triple band having lowpass and passband filter constructed on several crumpled thin lines and stubs having three radials is presented in the literature^16^. Seven transmission zeros (TZs) provide for strong isolations, a large higher rejection band, and decent selectivity of passbands; nevertheless, the second bandpass passband’s insertion loss (RL) is comparatively high. A lowpass along with a tri-bandpass response filter has been designed and analyzed using a metamaterial-based inverted tee-shaped stub connected with an open circular ring^17^. It also consists complementary split ring resonator and a defected structure in the ground plane. In^18^, a lowpass filter with quad bandpass response was analyzed with the help of an altered tee-shaped resonator along with quarterly stub-loaded stepped impedance resonators. This filter suffers from poor transmission coefficients and large structural size.

As far as the author’s knowledge, no filter has yet been documented in scholarly works that can only offer pentaband having a low pass in addition to quad-band bandpass responses. In this research, a new, small-scale pentaband having lowpass and bandpass response with low loss of insertion and improved selectivity is presented. The suggested filter is examined through the creation of a prototype and subsequent measurement. The proposed filter has several advantages over earlier designs, including easy fabrication, small size, straightforward construction, low insertion loss, and excellent selectivity.

## Geometry

**Fig. 1:**
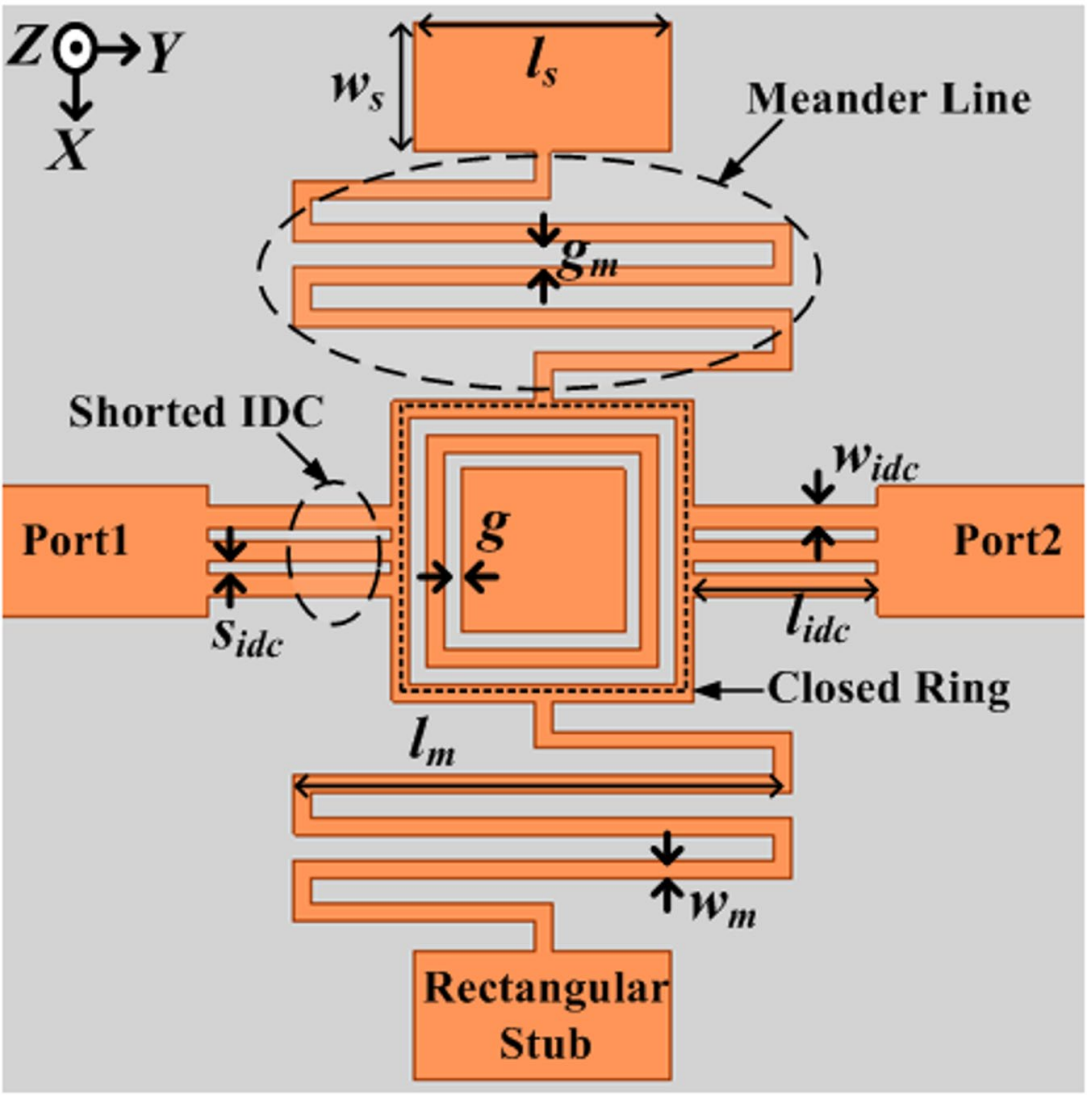
Configuration layout of the designed lowpass and quad passband filter. (All structural metrics are displayed in mm: *l*_*m*_ =11.6, *l*_*idc*_ =4.3, *l*_*s*_ =6.0, *w*_*m*_ = 0.4, *w*_*idc*_ = 0.5, *w*_*s*_ = 3.0, *S*_*idc*_ = 0.3, *g*_*m*_ = 0.6 and *g* = 0.6).

**Fig. 2:**
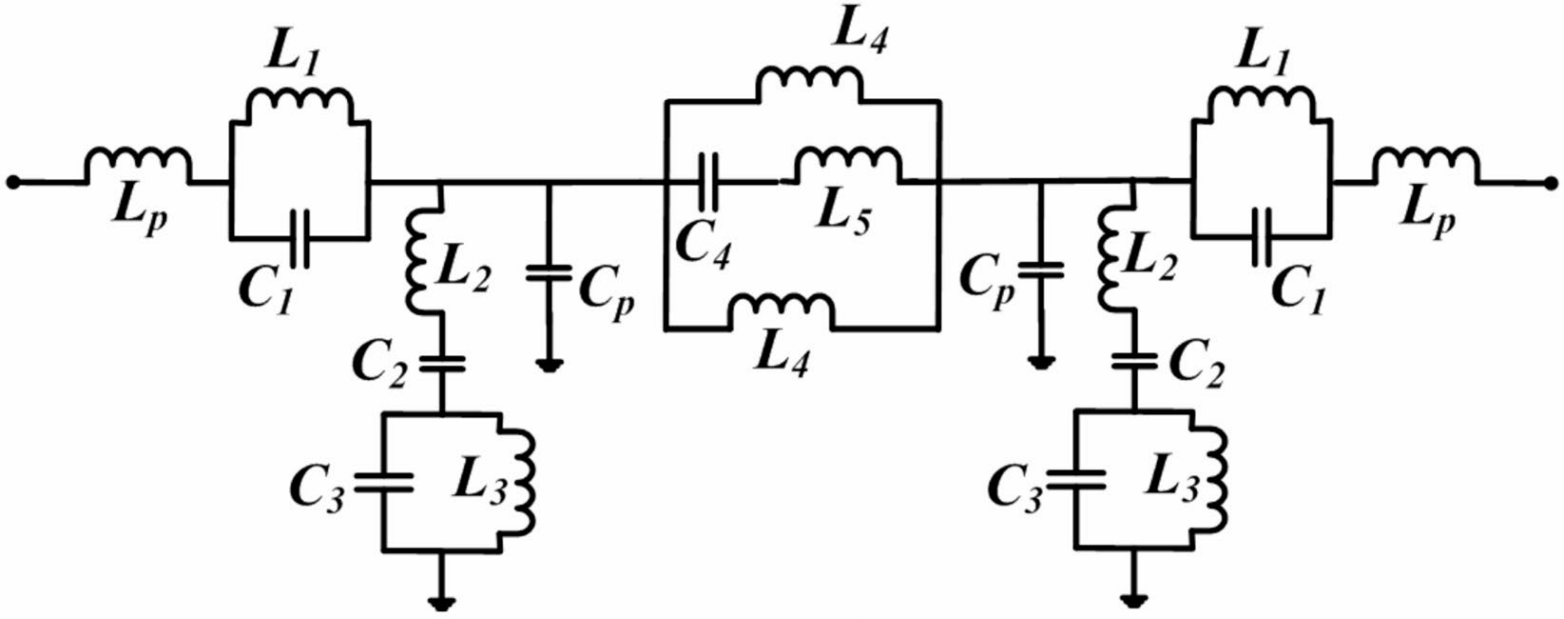
The comparable circuit of the presented filter.

The proposed filter comprises two sets of meander lines connected with rectangular stubs, a pair of shorted interdigital capacitors (IDC), and a closed ring. FR4 glass epoxy with a dielectric constant of 4.4, a tangent of loss (tanδ) value of 0.02, and a depth of 1.6 mm is employed as a substrate to apprehend the designed filter. Figure 1 shows the layout of the developed penta-band filter having lowpass and bandpass response with its optimum design dimensions. An intelligible LC comparable circuit diagram of the suggested structure is developed to analyze its performance, and it is displayed in Fig. 2. Here inductance *L*_*p*_ originates due to magnetic flux on feed line port-1/port-2. The parallel combination of capacitance *C*_*1*_ and inductance *L*_*1*_ in series is caused by shorted IDC. In shunt, the series combination of inductance capacitance *L*_*2*_*C*_*2*_ appears due to the meander line and because of the rectangular stub tank circuit *L*_*3*_*C*_*3*_ generated. The parasitic capacitance known as *C*_*p*_ is connected to the voltage gradient that exists between the copper’s upper and bottommost layers. Additionally, the parallel grouping of two inductance *L*_*4*_, inductance *L*_*5*,_ and capacitance *C*_*4*_ is attributed to the closed rings having some gaps between them.

**Fig. 3:**
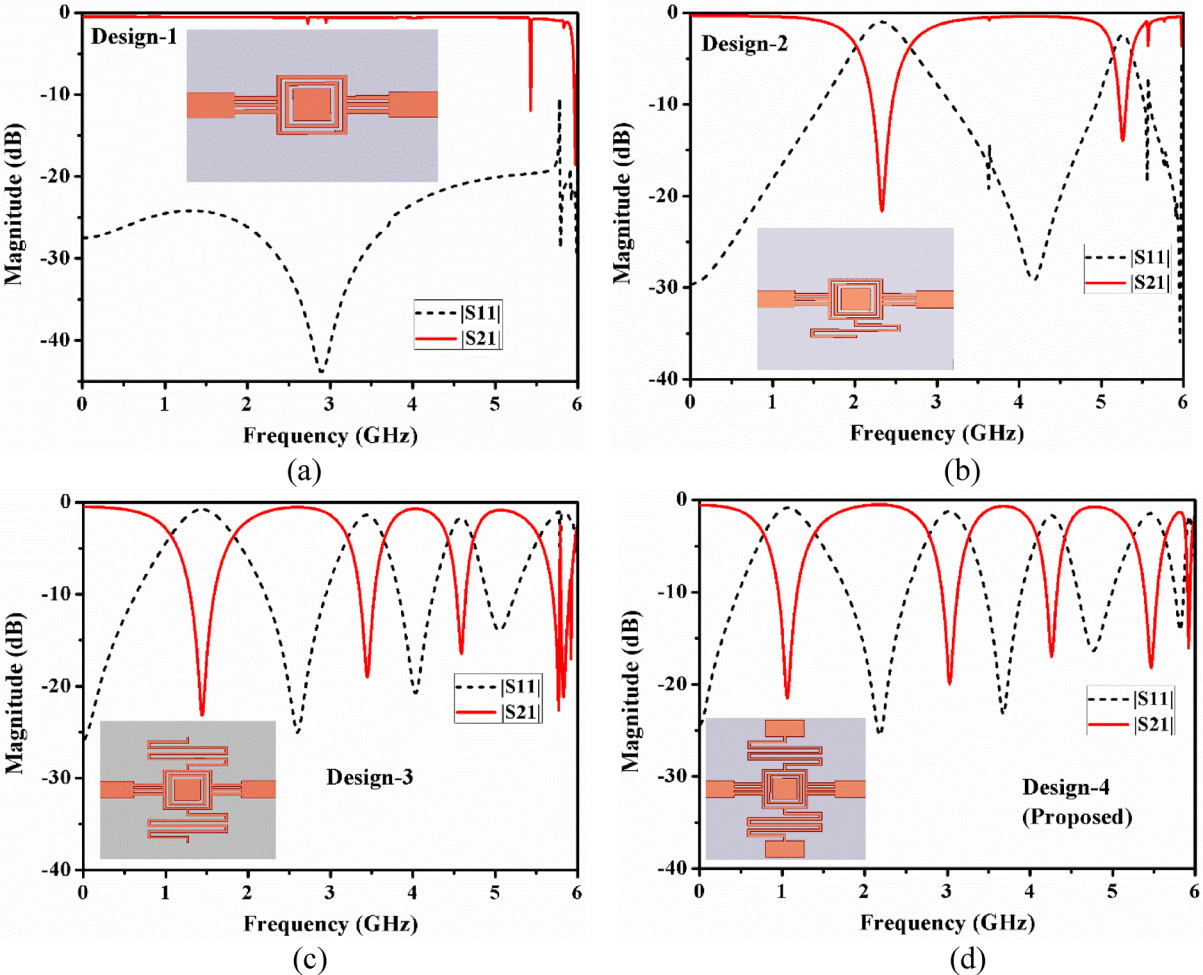
Specific development stages for the suggested quad-band filter: (**a**) Design-1, (**b**) Design-2, (**c**) Design-3, and (**d**) Design-4 (proposed).

By the Keysight Advanced Design System (ADS) circuit simulator, Fig. 2 is verified in terms of their resonating frequencies, and the lumped parameter values that are retrieved as: *L*_*p*_ = 19.1, *L*_*1*_ = 0.12, *L*_*2*_ = 3.1, *L*_*3*_ = 5.3, *L*_*4*_ = 1.3, *L*_*5*_ = 5.1, *C*_*p*_ = 2.64, *C*_*1*_ = 10.3, *C*_*2*_ = 1.5, *C*_*3*_ = 1.05, *C*_*4*_ = 1.05. All inductance and capacitance values are in nH and pF respectively. Figure 3 illustrates the ample design steps of the suggested filters using their sub-structures and their associated transmission and reflection performances. The development process of the presented lowpass and quad passband filter is divided into four major steps and these steps are concise: (i) Design-1: Initially a lowpass filter is developed and depicted in Fig. 3(a). This structure consists of closed ring resonators and, a shorted interdigital capacitor, connected with feed lines port-1 and port-2 on top of the substrate, and at the bottom consists metallic copper layer. It provides poor transmission characteristics. (ii) Design-2: Additional meander line at one side of the closed ring to get passband due to inclusion of a series combination of LC in the shunt. As an outcome, a lowpass having a 3-dB cut-off frequency of 2.0 GHz and one passband from 2.7 to 5.0 GHz has been obtained, depicted in Fig. 3(b). (iii) Design-3: To get the higher number of passbands two sets of doubled meander line structure was added in both side of closed rings, other two sides are conned with shorted IDC and feedlines. As a result, a lowpass and the triple bandpass response has been achieved, illustrated in Fig. 3(c). (iv) Design-4: To increase the higher number of passband two rectangular stubs have been added in meander lines, provides a tank circuit in the shunt. Which will be able to increase one more passband and provide better impedance matching. The proposed structure named Dsign-4, is depicted in Fig. 3(d), which provides one lowpass and quad-bandpass response. It was noticed that it shows lowpass 3-dB cut-off frequency fc = 0.75 GHz, and quad passband responses (1.39–2.78/3.26–4.09/4.42–5.24/5.64–5.89). Additionally, in all bandpass bands, there has been a noticeable improvement in the transmission and return loss characteristics.

## Results and analysis

**Fig. 4:**
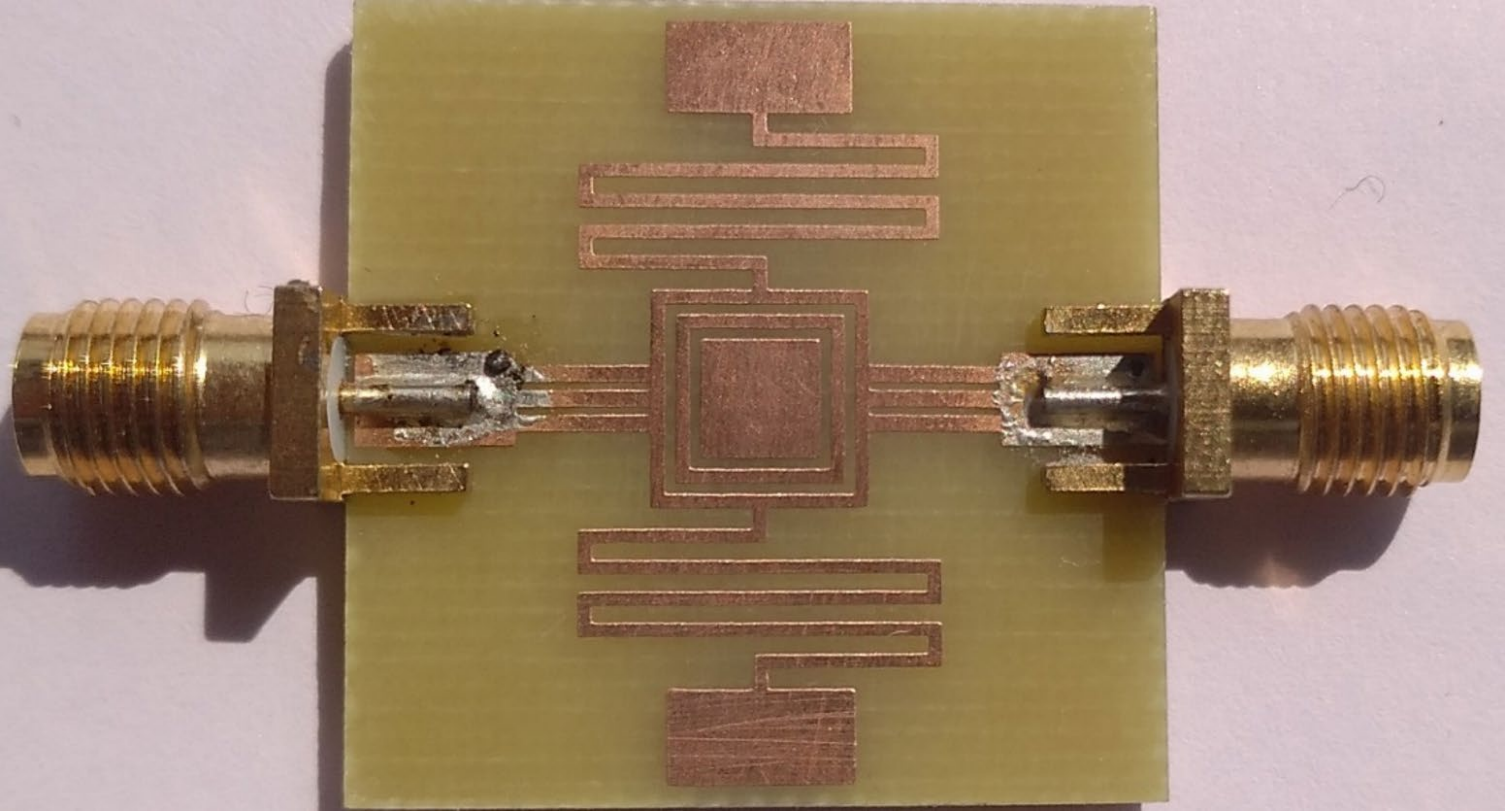
Prototype of proposed filter.

**Fig. 5:**
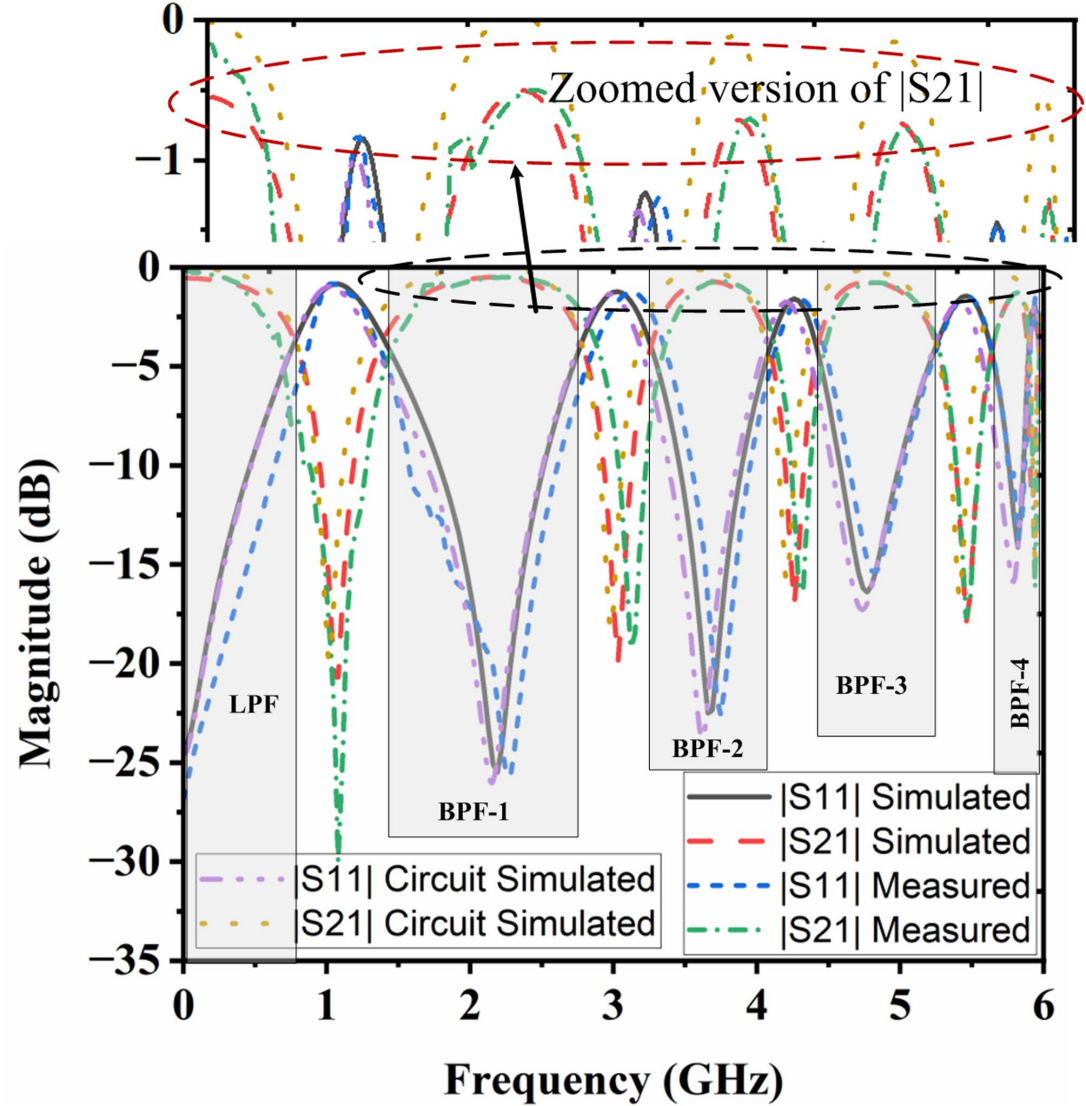
Experimental and simulated scattering parameters of the presented filter.

The manufactured model of the intended penta-band as a lowpass and passband filter is shown in Fig. 4. Afterwards, the master MS2038C hand-held Anritsu network vector analyzer is used to measure scattering parameters. Figure 5 compares measured, simulated and equivalent circuit simulated reflection and transmission properties. It can be observed that, except for a slight variation in the responses, the experimental data closely match the simulated results. The frequency shift can be attributed to connection losses, faulty soldering, and fabrication tolerance. The lowpass passband’s measured cut-off frequency (*f*_*c*_) at 3dB is 0.75 GHz. The average loss of insertion is found to be 0.4 dB within the lowpass response. The quad passband has transmission coefficients (S21) 0.4/0.5/0.6/1.0 dB at operating frequencies of 2.18/3.68/4.78/5.82 GHz, respectively. In full operational bandpass bands, the reflection coefficients (S11) values are 25.5/23.3/17.3/15.1 dB at their resonating frequencies respectively. Four bandpass responses have reported fractional bandwidths of 63.76% (1.39–2.78 GHz), 22.55% (3.26–4.09 GHz), 17.15% (4.42–5.24 GHz), and 4.3% (5.64–5.89 GHz) at operating frequencies of 2.18, 3.68, 4.78 and 5.82 GHz, respectively. The overall five transmission zeros are found at 1.05, 3.02, 4.24, 5.44, and 5.91 GHz.

**Fig. 6:**
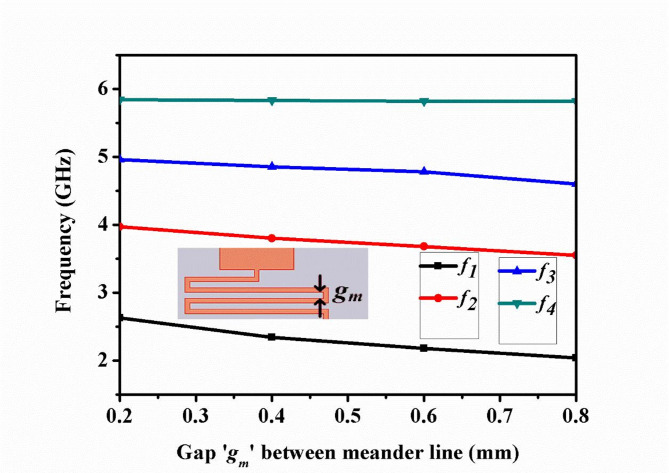
Change in the operating frequency of passbands due to variation of the gap between meander line ‘*g*_*m*_’.

**Fig. 7:**
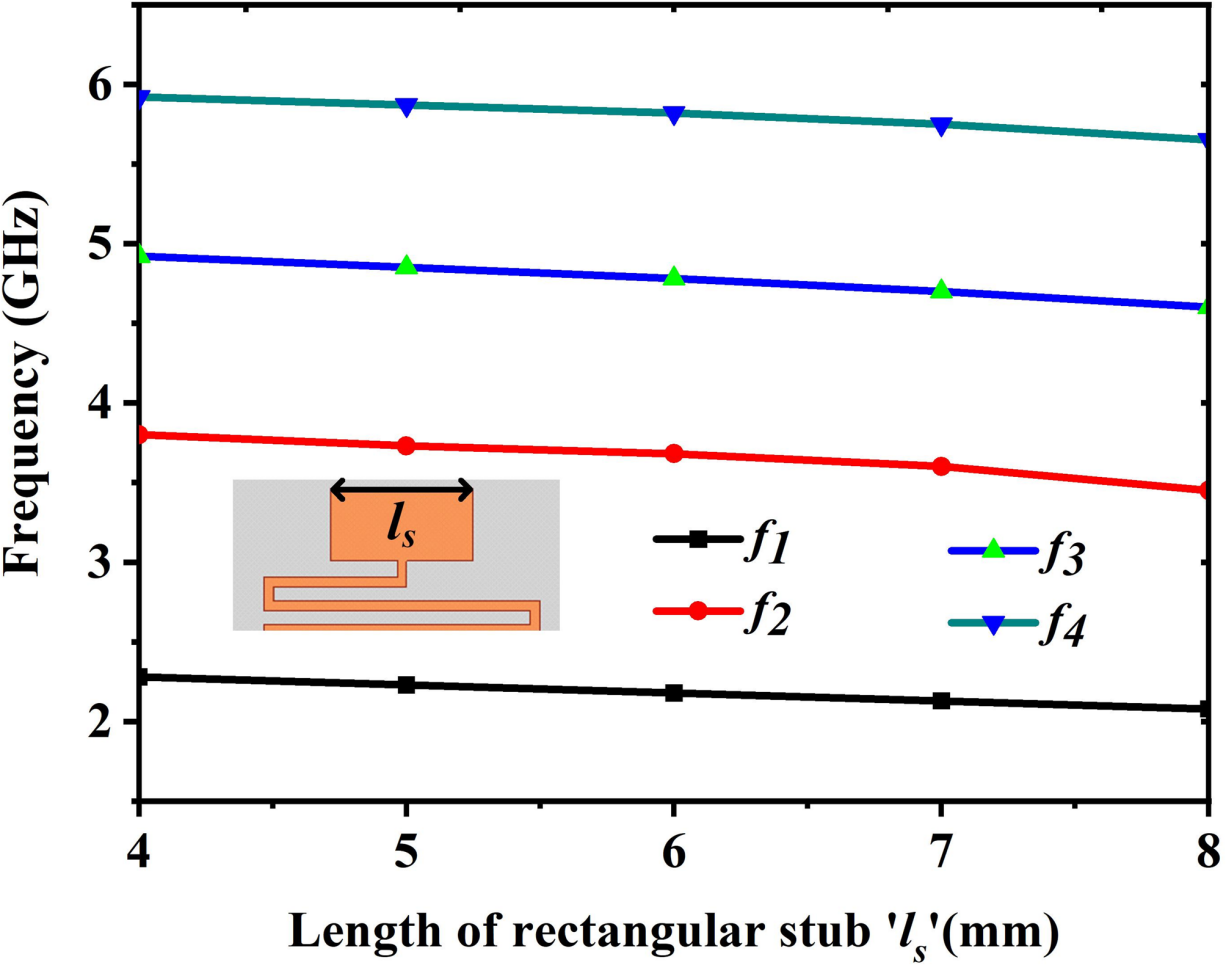
Change in the operating frequency of passbands due to variation of the length of rectangular stub ‘*l*_*s*_’.

The selectivity of lowpass is 95 dB/GHz, the and lower selectivity of first bandpass response is 55dB/GHz and 78 dB/GHz. The lower and upper selectivity of second, third and fourth passbands are 77dB/GHz, 100 dB/GHz, 120 dB/GHz, 78 dB/GHz, 320 dB/GHz, respectively. The variation of the operating frequency of the quad passband because of the gap (*g*_*m*_) between the meander line is illustrated in Fig. 6. As the value of ‘*g*_*m*_’ increases the operating frequency of the first, second, and third passbands namely *f*_*1*_, *f*_*2*_, and *f*_*3*_, respectively decreases towards the lower value of frequency. The operating frequency of the fourth passband *f*_*4*_ remains constant with a change in the value of ‘*g*_*m*_*’*. The variation of the operating frequency of the quad-band bandpass with respect to variation of length of rectangular stub ‘*l*_*s*_’, shown in Fig. 7. As the length ‘*l*_*s*_’ increases, inductance value of rectangular stub virtual ground increases, the operating frequencies decreases towards lower value.

Roll-off factor (ROF), often known as selectivity, is a crucial parameter of any filter. It is the rate where the transmission coefficient varies from the bandpass to the bandstop. A large value of selectivity is essential for filters since it demonstrates the filter’s capacity to accept some frequency regimes rapidly and reject others. The attenuation slope of lowpass transmission falls from − 3dB to − 21.5 dB with a frequency range of 0.75–1.05 GHz, corresponding to ROF 64.1 dB/GHz. Similarly, for the first, second, third, and fourth passbands, correspondingly, the left skirt selection ROF characteristics are 57.8, 28.3, 87.5, and 88.2 dB/GHz, and the right selection of skirt features are 77.2, 87.5, 60.0, and 325.1 dB/GHz. The figure of merit (FOM) displays the lowpass filter’s overall performance. High selectivity and strong out-of-band rejection characteristics, which are necessary for filter performance, are demonstrated by the greater FOM value. Its value is found with the following expressions^13^:

1 $$\:FOM=\frac{RSB\times\:\xi\:\times\:SF}{NS\times\:AF}$$

1a $$\:RSB=\frac{Stop\:band\_bandwidth}{Stop\:band\_centre-frequency}$$

1b $$\xi =\frac{{{a_{\hbox{max} }} - {a_{\hbox{min} }}}}{{{f_s} - {f_c}}}dB/GHz$$

1c $$NS=\frac{size\:(length\:\times\:width)}{{\lambda\:}_{g}^{2}}$$

where, RSB represents the relative bandwidth of the stopband, selectivity (ξ), normalized size of the structure (NS), ${a_{\hbox{min} }}$ and ${a_{\hbox{max} }}$shows 3dB and 20 dB point of attenuation, *f*_*s*_ represents 20 dB frequency of stopband, *f*_*c*_ displays the frequency of cutoff at 3dB and *λ*_*g*_ displays wavelength guided at a cut-off of low pass response. The planned lowpass gives a selectivity of 64.1 dB/GHz, RSB 0.61, NS of 0.04 *λ*_*g*_ × 0.06 *λ*_*g*,_ and FOM of 23,498. Table 1 summarises comparisons between the designed lowpass-quad passband filter with the related filter circuits. This consists of the frequency of lowpass cut-off *f*_*c*_, bandpass resonating frequencies *f*_*0*_, insertion loss at lowpass region, and passband response. It also comprises the dielectric constant ($\:{\epsilon\:}_{r}$) of uthe sed substrate, its loss tangent ($\:\text{tan}{\updelta\:}$) and the thickness of substrate *h*_*sub*_. Additionally, it compares circuit electrical size at the cut-off frequency of lowpass. The suggested circuit offers several advantages over the earlier research, including small size, good selectivity, and straightforward circuit design.

**Table 1:** Comparison of the proposed filter with similar comparative lowpass-bandpass filters.

Reference, year	f_c_ (GHz)/f_0_ (GHz)	Insertion loss of lowpass/bandpass response (dB)	$\:{\epsilon\:}_{r}$, $\:\text{tan}{\updelta\:}$, h_sub_ (mm)	Circuit size at f_c_ (λ_g_ × λ_g_)
^13^, 2020	1.0/1.9, 3.0	0.3/0.9, 0.8	10.2, 0.0023, 1.27	0.13 × 0.06
^2^, 2019	1.0/2.42, 5.37	0.1/0.69, 1.38	–	0.06 × 0.06
^15^, 2021	2.3/3.0, 6.2	0.2/0.8, 1.2	2.2, 0.0004, 0.508	0.26 × 0.35
^17^, 2023	1.11/1.62, 3.49, 4.51	< 0.4/0.52, 0.39, 0.65	10.2, 0.0023, 1.27	0.17 × 0.05
^18^, 2022	1/2.5, 4, 5.7, 7.3	< 0.8/2.5, 2.2, 2.59, 2	3.55, 0.0027, 0.508	–
^19^, 2023	1.2/2.42	0.1/1.1	3.58, 0.006, 0.508	0.218 × 0.218
^20^, 2023	0.92/1.7	0.1/0.05	9.67, –, 0.5	0.139 × 0.046
This work	0.75/1.18, 3.68, 4.78, 5.82	< 0.4/0.4, 0.5, 0.6, 1.0	4.4, 0.02, 1.6	0.04 × 0.06

## Conclusion

This paper represents, a unique small-scale penta-band lowpass-quad passband bandpass filter using a shorted interdigital capacitor, closed ring resonator, meander line, and rectangular-shaped virtual ground plane is examined. The main characteristics of the filter that is being described are low loss of insertion, compact size, wide bandwidth, and better figure of merit, because of the existence of a robust and vialess planar design. The proposed filter provides a lowpass passband at 3-dB cut-off frequency 0f 0.75 GHz with the downsized electrical size of 0.04*λ*_*g*_ × 0.06*λ*_*g*_. The filter’s attractive electromagnetic properties are thought to make it a promising tool for a variety of applications, with FR1 range of 5G wireless communication covering 0.45–6.0 GHz. It covers frequency band and fractional bandwidth of (1.39–2.78 GHz), 22.55% (3.26–4.09 GHz), 17.15% (4.42–5.24 GHz), and 4.3% (5.64–5.89 GHz).

## Data Availability

No datasets were generated or analysed during the current study.
